# PARV4: An Emerging Tetraparvovirus

**DOI:** 10.1371/journal.ppat.1004036

**Published:** 2014-05-01

**Authors:** Philippa C. Matthews, Amna Malik, Ruth Simmons, Colin Sharp, Peter Simmonds, Paul Klenerman

**Affiliations:** 1 Nuffield Department of Medicine, Peter Medawar Building for Pathogen Research, Oxford, United Kingdom; 2 Department of Infectious Diseases and Microbiology, Oxford University Hospitals NHS Trust, John Radcliffe Hospital, Oxford, United Kingdom; 3 Department of Paediatrics, Peter Medawar Building for Pathogen Research, Oxford, United Kingdom; 4 The Roslin Institute, The University of Edinburgh, Easter Bush, Midlothian, Scotland, United Kingdom; 5 NIHR Biomedical Research Centre, John Radcliffe Hospital, Headington, Oxford, United Kingdom; University of Florida, United States of America

## What Is PARV4?

PARV4 was first reported in 2005 in a hepatitis B virus–infected injecting drug user (IDU) [1]. It was detected by a screening process that aimed to identify new DNA viruses in subjects reporting risk factors for HIV combined with nonspecific symptoms of “viral infection syndrome”, including fatigue, malaise, and headache [1].

**Figure 1:**
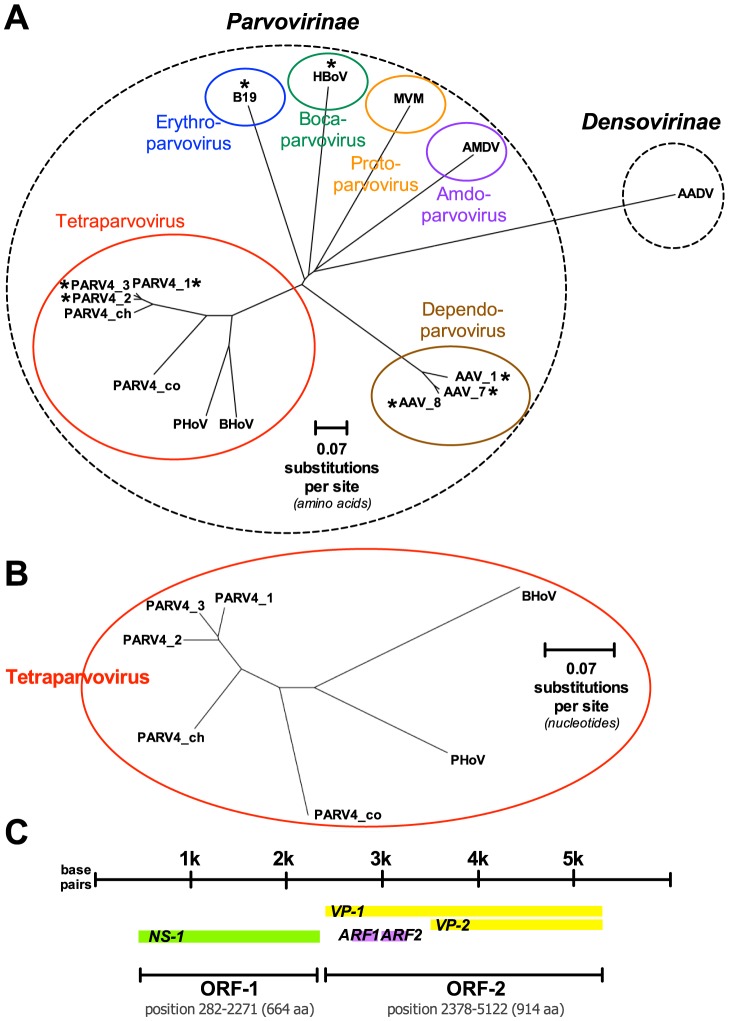
Genetics and genomics of PARV4. (A) Neighbour-joining phylogenetic tree reconstructed using amino acid sequences of ORF-1 (nonstructural protein) of Parvovirinae species. Sequences downloaded from NCBI (https://www.ncbi.nlm.nih.gov/genbank/). Sequences were selected based on completeness of sequence (minimum 621 amino acids per sequence), date of publication (all published between 2002 and 2010), and to provide representative viruses within each of the major Parvovirinae genera. Alignments and tree reconstruction were performed using Clustalw2 (http://www.ebi.ac.uk/Tools/msa/clustalw2). Subfamilies Densovirinae and Parvovirinae are indicated by dashed ovals. Genera within Parvovirinae are indicated by solid-coloured ovals and labelled with newly proposed genus nomenclature [23]. Viruses known to cause human infection are marked ‘*’. PARV4 genotypes 1–3 are in a new genus variously termed *Partetravirus* or *Tetraparvovirus*, together with animal parvoviruses (bovine and porcine hokovirus and PARV4-like viruses infecting nonhuman primates [8]) to which they are most closely related [2], [23]. Individual taxa are as follows, with GenBank ID in square brackets: PARV_1 = PARV4 genotype 1 [ACD71480.1]; PARV_2 = PARV4 genotype 2 [ABV71690.1]; PARV_3 = PARV4 genotype 3 [ACF94533.1]; PARV4_ch = PARV4-like virus of chimpanzees [AFD01617]; PARV4_co = PARV4-like virus of colobus monkey [AFD01599]; PHoV = porcine hokovirus [ADN44557.1]; BHoV = bovine hokovirus [ABY67685.1]; AAV_1 = adeno-associated virus 1 [AAU05367.1]; AAV_7 = adeno-associated virus 7 [YP_077177.1]; AAV_8 = adeno-associated virus 8 [YP_077179.1]; B19 = human parvovirus B19 [ABC87246.1]; AMDV = Aleutian mink disease virus [ACY54678.1]; MVM = minute virus of mice [ABB01353.1]; AADV = *Aedes aegypti* densovirus [YP_002854229.1]. Percentage amino acid sequence identity for tetraparvoviruses in comparison to PARV_1, calculated for NS1 protein using ClustalW2, are as follows: PARV4 _2, 96.4%; PARV4_3, 97.0%; PARV4_ch, 91.5%; PARV4_co, 67.8%; BHoV, 59.5%; PHoV, 58.1%. (**B**) Neighbour-joining phylogenetic tree reconstructed using full-length nucleotide sequences of species within the Tetraparvovirus subfamily. Methods and individual isolates as for Figure 1A. Percentage nucleotide sequence identity in comparison to PARV_1, calculated for full-length sequence using ClustalW2, are as follows: PARV4 _2, 92.1%; PARV4_3, 92.9%; PARV4_ch, 83.3%; PARV4_co, 71.3%; BHoV, 65.1%; PHoV, 65.3%. (**C**) Schematic diagram of PARV4 genome. Diagram based on NCBI Reference Sequence: NC_007018.1 [1]. Open Reading Frame 1 (ORF-1) encodes Non-Structural Protein 1 (NS-1); this region is responsible for potentially cytopathic effects of the virus [2]. Open Reading Frame 2 (ORF-2) comprises overlapping proteins Viral Protein 1 (VP-1) and Viral Protein 2 (VP-2), which encode structural capsid proteins. Protein lengths are shown as number of amino acids (aa). Additional Reading Frames (ARFs) are conserved across PARV4 genotypes; shown as ARF-1 (67 amino acids) and ARF-2 (86 amino acids) [5].

PARV4 belongs to the Parvovirus family, characterised by small, non-enveloped, single-stranded DNA viruses, with an icosahedral capsid. Parvoviruses infect a diverse range of hosts, and are divided into subfamilies Parvovirinae and Densovirinae, infecting vertebrates and arthropods respectively. PARV4 is one of only four groups of parvovirus known to infect humans, the others being parvovirus B19, human bocavirus, and adeno-associated viruses (Figure 1A, 1B).

The genome of PARV4 is approximately 5 kB long and comprises two open reading frames (ORFs), encoding three genes (Figure 1C). ORF-1 encodes a nonstructural protein (NS-1) essential for viral replication and also potentially responsible for cytopathic effects, as it can induce cell cycle arrest in vitro [2]. ORF-2 divides into two overlapping structural proteins, viral capsid proteins 1 and 2 (VP-1 and VP-2; Figure 1C). There is no in vitro culture system for PARV4, and it is unknown whether the virus can replicate autonomously.

## What Is the Origin of PARV4 in Humans?

PARV4 isolates have been subclassified into three genotypes (Figure 1B). Genotypes 1 and 2 (the latter originally termed PARV5) are predominant in Europe, North America, and Asia [3], [4]; genotype 3 is most widespread in Africa [5], [6]. Genetic diversity within each genotype is minimal, leading to one possible inference that the spread of each has been a relatively recent phenomenon, likely originating within the past 20–30 years [4]. It is possible that the three genotypes represent separate zoonotic transmissions of PARV4 into human populations, perhaps from chimpanzees and monkey species that harbour the most closely related parvoviruses to PARV4 [4], [7]. However, in a recent study [8], the nonhuman PARV4-like variants were species-specific, despite frequent opportunities for transmission, including blood contact, between nonhuman primates and human hunters. Despite its scarcity in Western countries, PARV4 may, alternatively, represent the human lineage of a parvovirus that has remained species-specific throughout the evolution of the *Partetravirus* genus.

## Who Gets PARV4 and How Is It Transmitted?

### (1) Evidence for parenteral and vertical transmission

To date, the best evidence about PARV4 transmission comes from IDU cohorts in Europe and North America, in which PARV4 is strongly associated with hepatitis C virus (HCV) and HIV infection: up to 95% of individuals with these viruses are positive for PARV4 IgG [9]. Likewise, in China, PARV4 infection has been strongly associated with the presence of either chronic hepatitis B virus (HBV) or HCV [3], and in a United Kingdom autopsy series, PARV4 DNA was found only in subjects coinfected with HIV [4].

However, parenteral transmission is also clearly possible independently from other blood-borne viruses, and PARV4 IgG has been reported in the absence of HIV, HBV, or HCV in the IDU population [10], in haemophilia patients [11], and in patients with a history of intra-muscular injections [12]. The potential for placental transmission has also been documented in a small series from Taiwan, in which neonates with PARV4 viraemia were born to IgM positive mothers [13].

PARV4 infections in European or North American populations are rare in individuals without risk factors for blood-borne viruses, suggesting little or no transmission in the general population. Indeed, even within households of PARV4-positive haemophiliacs, contacts are IgG negative [11].

### (2) Evidence for non-parenteral transmission

Alternative transmission routes are suggested by cohorts in which the background population has a higher prevalence of PARV4 IgG antibodies, and by PARV4-positive individuals who lack risk factors for parenteral transmission. One North American study, surprisingly, found PARV4 in 2% of plasma samples [14], although this prevalence should be interpreted with caution, because the risk factors for blood borne viruses in donors are not well characterised. In this study, fluctuations in the rate of PARV4 were postulated to reflect seasonal variation or bouts of epidemic transmission [14].

Even higher rates of PARV4 IgG seropositivity are reported in different geographic locations, strongly suggesting non-parenteral transmission. Up to 22% of HBV/HCV-negative individuals in a Chinese cohort were IgG positive [3], and around one in three adults in a variety of sub-Saharan African locations is positive in the absence of other blood-borne viruses [15]. Although a proportion of these cases may be parenterally transmitted (probably relating to iatrogenic exposures) [12], others may be acquired by alternative routes—although identifying these is problematic. A study of West African children with respiratory or gastrointestinal symptoms revealed the presence of PARV4 DNA in 0.5–0.8% of nasal and faecal specimens, suggesting the potential for either respiratory and/or faeco-oral transmission [16]. In another study of Ghanaian children, demographic factors were identified as markers of infection risk, including lack of access to a kitchen and living close to a river [6].

## Does PARV4 Cause Clinical Symptoms?

There is currently no definitive clinical syndrome associated with PARV4 infection, and the potential pathogenicity of related hokoviruses in animals is also unknown [17]. In the majority of instances, PARV4 viraemia appears to be self-limiting and asymptomatic [6], and there is no consistent association with increased severity of co-existing blood-borne viruses [3].

However, in a minority of reports, a range of possible disease outcomes are described in individuals with evidence of past or current PARV4 infection, including respiratory or gastrointestinal symptoms, hepatitis, rash, and encephalitis (Table 1). Notably, most of these studies describe small numbers of patients, and none is definitively able to attribute clinical manifestations to the presence of PARV4. Establishing cause and effect is further confounded by the close relationship between PARV4 and other blood-borne viruses; for example, although a statistical correlation has been described between PARV4 positivity and early features of AIDS, this association is potentially confounded by the close relationship between PARV4 and both HCV status and individuals with a history of IDU [9].

**Table 1:** Clinical symptoms reported in subjects with PARV4 infection.

Reference	Characteristics and location of subject(s) with PARV4 infection^a^	Method of laboratory detection of PARV4 infection	Presenting clinical symptoms(s)
Benjamin et al., 2011 [21]	N = 2; children aged 2–3 years with suspected CNS infection; India.^b^	PARV4 DNA in CSF	Presumed encephalitis (fever and generalised convulsions).
Chen et al., 2011 [13]	N = 6; mother-infant pairs with nonimmune idiopathic hydrops in foetus; Taiwan.	Infants: five of six had PARV4 DNA in plasma.Mothers: four of six had PARV4 IgM; two of six had PARV4 IgG	Foetal hydrops (≥2 of ascites, pleural/pericardial effusion, skin oedema, polyhydramnios). Two of six babies died.
Drexler et al., 2012 [16]	N = 13; Children with respiratory or gastrointestinal symptoms; Ghana.^c^	PARV4 DNA in nasal secretions (N = 8, median age 32 months) or faeces (N = 5, median age 43 months).	Upper/lower respiratory tract symptoms or gastrointestinal symptoms.
Jones et al., 2005 [1]	N = 1; homeless male IDU, Hepatitis B-positive, HIV-negative; United States.	PARV4 DNA in serum.	Fatigue, arthralgia, neck stiffness, pharyngitis, diarrhoea, vomiting, confusion, night sweats.
Sharp et al., 2012 [11]	N = 9; haemophilia patients aged 10–21 years seroconverting to PARV4 IgG positivity over a 5-year period (seven were already HIV-positive); HGDS cohort, US.	Conversion from PARV4 IgG negative to positive; two had transient positive PARV4 IgM. All were positive for PARV4 DNA in serum (viral titre <10^3^–10^10^ copies/ml)	Rash in three subjects, unexplained hepatitis (but minimal disturbance of LFTs at the time of PARV4 IgG seroconversion).
Simmons et al., 2012 [9]	N = 193; subjects from Swiss HIV Cohort Study (www.shcs.ch/).	PARV4 IgG positive.	Early HIV-related symptoms (CDC-B symptoms).
Vallerini et al., 2008 [22]	N = 1; patient with Wegener's Granulomatosis on long-term steroid therapy; Italy.^b^	PARV4 DNA in serum.	Fever, anaemia (with erythroid hypoplasia on bone marrow biopsy), post-infectious glomerulonephritis, subsequent multiorgan failure.

Papers are listed in alphabetical order by first author.

a Denominator presented is the number of individuals positive for PARV4 (extrapolated from total number of subjects studied in each paper), except for Simmons et al. [9], where denominator is number with HIV.

b Other infectious causes of the clinical syndrome were excluded.

c Other pathogens were also present which may have explained the clinical syndrome.

CNS = central nervous system; CSF = cerebrospinal fluid; IDU = injecting drug user; LFTs = liver function tests.

## How Is PARV4 Infection Diagnosed and Documented?

Evidence of PARV4 infection is most frequently detected by an ELISA for specific IgG antibody to VP-2 [11]. This response appears to be sustained over time, as with other parvovirus infections; weak or transient VP-2 IgM positivity has also been reported in acute infection [11].

PARV4 DNA may be isolated from plasma in acute infection, generally with low viral loads (e.g. ≤3×10^4^ copies/ml) [11], [18], although acute viraemia of up to 10^10^ copies/ml has been reported [11]. Asymptomatic viraemia was reported in 8% of children in a Ghanaian cohort [6]. Different studies have reported the duration of viraemia lasting from 30 days [10] up to a mean of 7 months [11]. However, recrudescence or reinfection could also explain these relatively prolonged durations of viraemia [18]. Despite these reports of isolation of PARV4 DNA from serum [10], [11], [18], this is generally uncommon, suggesting that immune containment is good even in immunocompromised hosts [4].

## Is PARV4 Cleared after Acute Infection?

Like other human parvoviruses, PARV4 has the potential for persistence: DNA can be extracted from tissue long (indeed, possibly life-long) after primary infection [4], [19]. This is also supported by a high frequency and magnitude of T-cell responses to PARV4 (detected in vitro by Interferon-gamma ELISpot assays) [20], similar to that which is seen in response to other chronic or latent viral infections—the best characterised examples being the herpesviruses CMV and EBV.

It is not known exactly which tissues are reservoirs for PARV4, either in the acute or chronic phases of infection. However, different studies have described the detection of PARV4 DNA from a variety of sites including blood, lymphoid tissue, bone marrow, liver, and central nervous system [1], [4], [21]. The relationship between possible tissue tropism and clinical disease is not well characterised: viral DNA may persist indefinitely, but this archived virus does not necessarily reflect local disease. Conversely, specific pathology does not clearly relate to viral replication in local tissues; for example, anaemia in acute infection was not associated with high viral titres in bone marrow [22].

## Should We Worry about PARV4 in the Long Term?

Despite the lack of consistent evidence for PARV4-mediated disease, there are several concerns about the implications of this virus.

Other viruses that make an interspecies jump from animals to humans have demonstrated the potential to cause severe human disease, the most notable recent examples being the SARS coronavirus and highly pathogenic species of influenza. Related animal parvoviruses have the potential to cause significant host-specific pathology (e.g., fever, foetal loss, and chronic immunosuppression in pigs [17]). It is therefore possible that parvoviruses that make their way into the human host will ultimately be responsible for a greater range of significant pathology. Furthermore, the parenteral route of PARV4 transmission and its high prevalence in subjects coinfected with other blood-borne viruses puts immunocompromised patients at particular risk of acquiring infection; these are also the individuals likely to be most susceptible to clinical manifestations.

Parvoviruses are potentially resistant to traditional viral inactivation methods employed for plasma-derived products [1], [11], [14]. In the Western world, these risks are mitigated by modern virus inactivation procedures that are likely to be effective against parvoviruses and by the increasing use of recombinant clotting factors instead of plasma-derived products. However, transfusion-mediated transmission remains a potential issue in developing countries. The extent of this concern is uncertain given the doubts over the clinical significance of PARV4 infection.

We are left with important unanswered questions. What are the potential outcomes of infection with PARV4? Are there really multiple different modes of transmission? How frequently does viral persistence occur, and does this matter to the host? Further work is urgently needed to improve our understanding of this emerging infection.
